# Pylephlebitis Caused by *Fusobacterium nucleatum* in a Septuagenarian Healthy Caucasian Male: Atypical Presentation of Lemierre's Syndrome

**DOI:** 10.1155/2022/5160408

**Published:** 2022-01-25

**Authors:** Muhammad Abbas, Monica Ioana Constantin, Akshay Narendra

**Affiliations:** ^1^Avalon University School of Medicine, Youngstown, OH, USA; ^2^Saint James School of Medicine, Park Ridge, IL, USA

## Abstract

Lemierre's syndrome (LS) is characterized by thrombophlebitis of the internal jugular vein caused primarily by *Fusobacterium necrophorum*. LS is usually suspected in fit young adults with prolonged or prior pharyngeal infection. Atypical Lemierre's syndrome is commonly defined as *Fusobacterium*-associated thrombophlebitis outside the head-neck veins and usually occurs in older patients than typical Lemierre's syndrome. Here we present a case of atypical LS in a septuagenarian healthy Caucasian male with no prior history of pharyngitis and in whom both computed tomography (CT) and magnetic resonance imaging (MRI) demonstrated partial portal vein thrombosis associated with *Fusobacterium nucleatum*. This case report confirms previous reports of *Fusobacterium nucleatum*-associated LS variants presenting with abdominal vein thrombosis and illustrates clinical recovery after a combination of anticoagulation and antibiotic therapy.

## 1. Introduction

Septic thrombophlebitis is the occurrence of venous thrombosis associated with bacteremia [1], which can be caused by throat infections, dental procedures, gingivitis, or central/peripheral catheterizations [2]. Suppurative thrombophlebitis of the portal vein is known as pylephlebitis. Historically fatal and identified only on autopsy, the discovery of antibiotics and improvements in imaging and diagnostic modalities has reduced the mortality of pylephlebitis down to 10–30% [3]. However, the non-specificity of its clinical presentation makes it difficult to accurately estimate the incidence of the disease [4]. The underlying focus of infection usually includes, but is not limited to, appendicitis, diverticulitis, pancreatitis, and peritonitis [5, 6]. The most common microorganisms contributing to pylephlebitis are *B. fragilis*, *E. coli*, and *Streptococcus* spp., among others [5, 6].

*Fusobacterium* sepsis, more commonly associated with thrombophlebitis of the internal jugular vein, also known as Lemierre's syndrome, is a rare cause of pylephlebitis or other forms of thrombophlebitis outside of the head-neck syndrome, especially in the gastrointestinal system. These conditions are sometimes referred to as atypical Lemierre's syndrome [7, 8]. In this report, we present the case of a *Fusobacterium*-associated thrombophlebitis of the portal vein in an otherwise healthy Caucasian male.

## 2. Case Presentation

A 73-year-old Caucasian man with a past medical history of hypertension, hyperlipidemia, and melanoma presented to the Emergency Department (ED) with several weeks of fever. He had visited Arizona with his family prior to the onset of symptoms. On his return, he had developed a non-productive cough, fever, cold sweats, and weight loss which prompted him to visit his primary care physician. He was treated with supportive care and had an outpatient chest/abdomen/pelvis non-contrast CT scan done which showed a hazy density in the mesentery and an increase in the number of small lymph nodes. The CT scan also showed prominent small bowel folds involving the jejunum and an 8 mm ground glass nodule in the right lung. He was seen in the ED the next day and was discharged with ciprofloxacin for pyuria. Coccidioidomycosis antibodies were found negative during this visit. He was referred to an Infectious Disease (ID) specialist and was prescribed fluconazole for empiric treatment of Valley Fever (coccidioidomycosis).

Due to persistent fever and failure to respond to empiric therapy of Valley Fever, the patient presented to the ED 12 days later where he was admitted to the hospital. His blood pressure (BP) was 125/61 mmHg, pulse was 108 bpm, oral temperature was 101.5°F (38.6°C), RR was 20 bpm, and SpO_2_ was 98% on room air. He was alert and oriented to person, place, time, and situation with all cranial nerves intact. Physical exam showed no lymphadenopathy and was otherwise unremarkable.

A review of complete blood count (CBC) on admission showed significant elevated white blood cell count (19.1 *∗* 10^9^ cells/L), elevated neutrophils (85.1%), low red blood cell count (3.71 million cells/mm^3^), hemoglobin (10.4 g/dL), low hematocrit (31.4%), and low lymphocytes (5.5%). Basic metabolic panel showed hyponatremia (130 mEq/L), hypokalemia (3.2 mEq/L), hypocalcemia (7.6 mg/dL), and low chloride (91 mEq/L). Liver function tests showed abnormal proteins (2+), low albumin (2.3 g/dL), and elevated alkaline phosphatase (163 U/L). Other blood work showed elevated lactate (2.3 mmol/L), erythrocyte sedimentation rate (92 mm/hr), and C-reactive protein (>300.0 mg/L). All other parameters for blood were unremarkable. Urinalysis was significant for protein (1+), bacteria (2+), white blood cells (6–10), and red blood cells (5–10). All other parameters were within normal limits. Urine bacterial culture and *Legionella* antigen were both negative.

Chest X-ray showed no evidence of pulmonary infiltrates. CT of abdomen/pelvis with contrast showed partial portal vein thrombosis extending into multiple peripheral portal structures within the liver and attenuation of the superior mesenteric vein (SMV) with possible thrombosis in the SMV as well. New diffuse moderate abdominal ascites was also noted. Abdominal ultrasound was significant for multiple echogenic liver lesions that warranted further evaluation via MRI. MRI with contrast of the abdomen confirmed portal vein thrombosis and intraperitoneal fluid. It also confirmed the presence of multiple hepatic lesions suggestive of possible hepatic abscesses given the patient's fever and leukocytosis. Blood smear was negative for plasmodium. A purified protein derivative (PPD) skin test for tuberculosis was negative. Stool cultures were negative for *Salmonella*, *Shigella*, *Campylobacter*, *Aeromonas*, *Plesiomonas*, and *Yersinia*. HIV-1 and HIV-2 were non-reactive. Blood cultures grew *Fusobacterium nucleatum*.

### 2.1. Treatment

While admitted to the hospital, the patient was started on anticoagulation with Lovenox (enoxaparin) 1 mg/kg every 12 hours as well as the antibiotics Zosyn (piperacillin/tazobactam), 3.375 g q6 hrs totaling 13.5 g (12 g piperacillin/tazobactam) and Flagyl (metronidazole). Metronidazole was later switched to doxycycline. Once stool cultures results returned negative, doxycycline was stopped. On discharge from the hospital, he was prescribed a daily dose of warfarin 5 mg anticoagulation and bridging with enoxaparin. Enoxaparin was discontinued when the INR reached therapeutic doses. He was also treated with ertapenem 1 g daily for 6 weeks at the infusion center. He was told to stop his other medications: lovastatin 40 mg and Dyazide (triamterene-hydrochlorothiazide (HCTZ)) 37.5–25 mg.

### 2.2. Outcome

In the weeks following discharge, the patient developed massive ascites and bilateral pitting edema of the legs. He was immediately referred to a hepatologist and was treated with diuretic therapy. The patient completed treatment and eventually made a full recovery in the subsequent months.

## 3. Discussion

A typical case of Lemierre's syndrome is initiated by a pharyngeal infection caused by *Fusobacterium necrophorum*, a Gram-negative bacillus. Pathogens colonize the area and then drain into the lateral pharyngeal space, where they approach the internal jugular vein [9] The first affected location within the pharynx is the peritonsillar tissue and its associated tonsillar veins. *F*. *necrophorum's* ability to aggregate platelets contributes to the formation of a thrombus within the tonsillar veins [10]. This ability, possibly combined with vessel compression by inflammation and edema, may result in lumen obstruction and loss of drainage/blood flow to the peritonsillar tissue. Local infection seeps into the internal jugular vein and causes similar sequela there. Furthermore, it can also lead to the development of septic thrombi that break off and travel throughout the systemic circulation causing both local and systemic infection and inflammation [9].

A mucosa already damaged by an infection may create an environment conducive for a *Fusobacterial* superinfection. In addition to a suggestive past medical history, patients at increased risk of developing Lemierre's syndrome are adolescents or young adults who were previously fit. Lemierre's syndrome is a specific form of septic thrombophlebitis, which includes venous thrombosis associated with local bacterial infection occurring at several anatomic locations [11]. Current literature describes a variant of Lemierre's syndrome involving an older demographic who present with an initial infection and thrombosis of the portal vein [8, 12]. The pathogenesis of the thrombus formation remains the same; however, the strain of *Fusobacterium* involved in the portal vein thrombosis differs from that of a typical case of Lemierre's syndrome. Typically, *Fusobacterium necrophorum* is involved, but in the atypical cases, the responsible pathogen points to *Fusobacterium nucleatum* [12]. In light of the recent report by Lazar et al., describing several cases of portomesenteric thrombosis due to *Fusobacterium necrophorum* infection [8], atypical Lemierre's syndrome may be attributed to *Fusobacterium* spp. infection.

Whether Lemierre's syndrome presents typically or atypically, all reported cases had fever and leukocytosis [10]. In an atypical presentation of Lemierre's syndrome, patients with pylephlebitis as the initial site of infection would show signs of fever, sweats, weight loss, and intermittent midepigastric or right upper quadrant pain in the early stages of clinical manifestations of signs and symptoms [13]. A typical presentation of Lemierre's syndrome, however, includes a sore throat and fever [9]. Associated symptoms include neck tenderness, swelling, and cervical lymphadenopathy related to the peritonsillar abscesses. As the infection spreads towards the jugular veins, we see unilateral tenderness and swelling at the mandibular angle (parallel with the sternomastoid muscle), known as the “cord sign.” This indicates internal jugular thrombosis and is present in 25% to 45% of cases [9]. In contrast to atypical Lemierre's syndrome, in typical Lemierre's syndrome, systemic inflammation and signs of multiorgan involvement as a consequence of septic emboli, which may also include gastrointestinal compromise, only occur in advanced stages of the disease.

This case report strengthens the existing literature related to variants of Lemierre's syndrome. It contributes to the main differences seen in cases of portal vein thrombosis secondary to *Fusobacterium* infection, i.e., *F. nucleatum* remains the main cause of infection and thrombosis in portal veins of elderly population. Evidence from a case report dating back to 2014 mentioned two cases involving portal vein thrombosis in context of *Fusobacterium* infections. The report mentioned that while *Fusobacterium* is known to induce platelet aggregation, thrombotic events outside of the head and neck are rare [8, 14]. When they do occur, they are mainly seen in elderly patients and mortality can approach 25% [8, 14].

In our case, the patient was an older male outside the typically younger age range of Lemierre's syndrome, and he had no prior infection of the head-neck area. To our knowledge, there are only six other reports of pylephlebitis associated with *F. nucleatum* bacteremia [8], of which four were associated with preceding oropharyngeal infection [13]. No information has been stipulated about the two remaining cases of *F. nucleatum*-induced pylephlebitis. Further investigations should emphasize on cases about *Fusobacterial* infections of the portal vein in previously fit individuals of an older age.

## 4. Conclusion

In summary, it is important for current literature to consider a variant of Lemierre's syndrome which causes pylephlebitis in its initial stages of infection, resulting from the colonization of the portal vein by a bacterium, that also belongs to the *Fusobacteria* species: *Fusobacterium nucleatum*. Pre-existing conditions or environmental factors are inconclusive in determining the reasons why a patient would develop an atypical case of Lemierre's syndrome, and further indications from current literature are insufficient to show evidence that supports a solid reasoning, as there are so few cases of *F. nucleatum*-induced pylephlebitis. However, regardless of its presentation, Lemierre's syndrome leads to widespread thrombosis and sepsis affecting many organ systems, and, to prevent these complications from developing, it needs to be identified rapidly and treated accordingly.

## Data Availability

The data used to support the findings of this study are available from the corresponding author upon request.

## References

[1] Mermel L. A., Allon M., Bouza E. (2009). Clinical practice guidelines for the diagnosis and management of intravascular catheter-related infection: 2009 update by the infectious diseases society of America. *Clinical Infectious Diseases*.

[2] Kar S., Webel R. (2014). Septic thrombophlebitis. *American Journal of Therapeutics*.

[3] Plemmons R. M., Dooley D. P., Longfield R. N. (1995). Septic thrombophlebitis of the portal vein (pylephlebitis): diagnosis and management in the modern era. *Clinical Infectious Diseases*.

[4] Mellor T. E., Mitchell N., Logan J. (2017). Lemierre’s syndrome variant of the gut. *BMJ Case Reports*.

[5] Kanellopoulou T., Alexopoulou A., Theodossiades G., Koskinas J., Archimandritis A. J. (2010). Pylephlebitis: an overview of non-cirrhotic cases and factors related to outcome. *Scandinavian Journal of Infectious Diseases*.

[6] Choudhry A. J., Baghdadi Y. M. K., Amr M. A., Alzghari M. J., Jenkins D. H., Zielinski M. D. (2016). Pylephlebitis: a review of 95 cases. *Journal of Gastrointestinal Surgery*.

[7] Zheng L., Giri B. (2016). Gastrointestinal variant of Lemierre syndrome. *American Journal of Therapeutics*.

[8] Lazar N., Sardarli K., Imam Z., Khasawneh M., Hader I. (2021). A rare twist of the forgotten disease: a case of Fusobacterium necrophorum sepsis with portomesenteric thrombosis and a review of the literature. *Case Reports in Gastrointestinal Medicine*.

[9] Valerio L., Zane F., Sacco C. (2021). Patients with Lemierre syndrome have a high risk of new thromboembolic complications, clinical sequelae and death: an analysis of 712 cases. *Journal of Internal Medicine*.

[10] Riordan T., Wilson M. (2004). Lemierre’s syndrome: more than a historical curiosa. *Postgraduate Medical Journal*.

[11] Valerio L. N., Riva N. (2020). Head, neck, and abdominopelvic septic thrombophlebitis: current evidence and challenges in diagnosis and treatment. *Hämostaseologie*.

[12] Moore J. A., Rambally S. (2017). Fusobacterium nucleatum bacteremia presenting with portal vein thrombosis: an abdominal Lemierre syndrome?. *The American Journal of Medicine*.

[13] Constantin J.-M., Mira J.-P., Guerin R. (2006). Lemierre’s syndrome and genetic polymorphisms: a case report. *BMC Infectious Diseases*.

[14] DePetrillo J. (2014). Two cases of portal vein thrombosis associated with Fusobacterium bacteraemia. *JMM Case Reports*.

